# Short-term outcomes after robot-assisted versus open radical cystectomy for bladder cancer in patients with diabetes mellitus: an analysis of the United States Nationwide Inpatient Sample of 2005-2018

**DOI:** 10.7150/ijms.102667

**Published:** 2025-01-01

**Authors:** Cho-Hsing Chung, I-Shen Huang, Wei-Tang Kao

**Affiliations:** 1 Department of Urology, Taipei Municipal Wan Fang Hospital, Taipei, Taiwan.; 2 Doctor of medicine., Taipei Medical University, Taipei, Taiwan.; 3 Department of Urology, Taipei Veterans General Hospital, Taipei, Taiwan.; 4 Department of Physiology, School of Medicine, National Yang Ming Chiao Tung University, Taipei, Taiwan.; 5 Department of Urology, College of Medicine, and Shu-Tien Urological Science Research Center, National Yang Ming Chiao Tung University, Taipei, Taiwan.; 6 Department of Urology, Shuang Ho Hospital, Taipei Medical University, Taipei, Taiwan.; 7 Doctor of medicine, Taipei Medical University, Taipei, Taiwan.

**Keywords:** bladder cancer, diabetes mellitus (DM), laparoscopic, nationwide inpatient sample (NIS), open radical cystectomy (ORC), robot-assisted radical cystectomy (RARC)

## Abstract

**Introduction**: Diabetes mellitus (DM) is associated with worse surgical outcomes, and is a risk factor for bladder cancer and subsequent oncological outcomes. This study evaluated outcomes robot-assisted radical cystectomy (RARC) compared to open radical cystectomy (ORC) in patients with DM.

**Materials and Methods**: Data of adults ≥ 18 years old with DM who underwent radical cystectomy were extracted from the United States National Inpatient Sample database 2005-2018. The outcomes were in-hospital mortality, prolonged length of stay (LOS), and postoperative complications.

**Results**: Data of 2,765 patients were analyzed. Patients who received RARC had a significantly lower odds of prolonged LOS (adjusted odd ratio (aOR) = 0.56, 95% CI: 0.45, 0.71), unfavorable discharge (aOR = 0.74, 95% CI: 0.56, 0.97), urinary complications (aOR = 0.75, 95% CI: 0.57, 0.98) and wound and device-related complications (aOR = 0.59, 95% CI: 0.41, 0.86) than ORC. Of patients < 70 years old, RARC was significantly associated with decreased odds for urinary complications (aOR = 0.59, 95% CI: 0.41, 0.84) and wound and device-related complications (aOR = 0.55, 95% CI: 0.32, 0.94) compared to ORC. In patients with a Charlson Comorbidity Index score of 0-1, RARC was associated with a lower risk of urinary complications (aOR = 0.74, 95% CI: 0.56, 0.98) and wound and device-related complications (aOR = 0.63, 95% CI: 0.43, 0.93) compared to ORC.

**Conclusions**: In patients with DM and bladder cancer, RARC appears to be associated with better short-term outcomes in terms of reduced risks of prolonged LOS, unfavorable discharge, urinary complications, and wound and device-related complications compared to ORC.

## Introduction

Bladder cancer is the most common malignancy of the urinary tract 1. In 2020, according to the Global Cancer Statistics Report approximately 575,000 individuals worldwide were diagnosed with bladder cancer, comprising 3% of all malignancies 2. Radical cystectomy, which can be done through open surgery or minimal invasive surgery, is the standard surgical treatment for non-metastatic muscle-invasive bladder cancer 3. It has been recognized that minimal invasive surgery is associated with lower risk of various short-term complications and mortality than open radical cystectomy (ORC) for bladder cancer 4.

Diabetes mellitus (DM) is a serious, long-term condition with a major impact on the lives and well-being of individuals, families, and societies worldwide 5. It is also a rising epidemic, and one of the leading causes of death worldwide 6. Patients DM may represent a unique population within the context of various health conditions, including greater risks for cardiovascular disease and a variety of cancers 7, 8. It has been documented that DM is associated with an increased risk for bladder cancer 9, 10. Further, previous studies have shown that DM is linked to worse survival rates across various cancers, including bladder cancer 11, 12. Moreover, DM is considered a comorbidity in patients undergoing surgery, and can complicate surgical procedures and have negative impact on postoperative outcomes 13.

Many studies have investigated and compared the effectiveness between robot-assisted radical cystectomy (RARC) and ORC for bladder cancer with respect to oncological and functional outcomes 4, 14-16. However, there has been limited research has focused specifically on patients with DM. Considering the significance of comprehending the surgical results among high-risk patients such as those with DM to facilitate clinical decision-making and enhance patient care, our study aims to assess the in-hospital outcomes following RARC as compared to ORC. To accomplish this, we utilized a nationally representative cohort from the United States (US).

## Methods

### Data source

This population-based, retrospective observational study extracted data from the United Stated (US) National Inpatient Sample (NIS) database. The NIS is the largest all-payer, continuous inpatient care database in the United States, and includes about 8 million hospital stays each year 17. The database is administered by the Healthcare Cost and Utilization Project (HCUP) of the US National Institutes of Health (NIH). The patient data consist of primary and secondary diagnoses, primary and secondary procedures, admission and discharge status, patient demographic information, projected payment source, hospital stay duration, and hospital characteristics (i.e., bed size/location/teaching status/hospital area).

Initial consideration is given to all hospitalized patients for inclusion. The continuously updated, annual NIS database contains patient information from around 1,050 hospitals in 44 states, representing a stratified sample of 20% of US community hospitals, as defined by the American Hospital Association.

### Study population

Hospitalized adults ≥ 18 years old with DM who underwent radical cystectomy between 2005 and 2018 were identified through the International Classification of Diseases, Ninth Revision and Tenth Revision, Clinical Modification (ICD-9-CM and ICD-10-CM) codes. Exclusion criteria were patients with incomplete data on main outcomes of interest, sex, and weight values of the dataset. Patients who underwent pure laparoscopic (i.e., without robot assistance) procedures alone were also excluded. Patients who received RARC and ORC were also identified through ICD-9 and ICD-10 procedure codes.

### Study outcomes

Outcomes were in-hospital mortality, prolonged length of hospital stay (LOS), postoperative complications, and unfavorable discharge (defined as discharge to a long-term care facility). In-hospital mortality information was identified from the discharge disposition. LOS of stay was calculated by subtracting the admission date from the discharge date. Postoperative complications included infection, urinary complications, acute kidney injury (AKI), and wound and device-related complication (i.e., complications or acute reaction that occur as a result of using surgical instruments or medical devices during procedures), and were identified through ICD codes.

### Covariates

Demographic data including age, sex, race, and family income-to-poverty ratio were extracted from the NIS database. Hospital-related characteristics (bed size and location/teaching status) were extracted from the database as part of the comprehensive data available for all participants, in accordance with other NIS studies in the medical literature.

### Statistical analysis

Since the NIS database covers a 20% sample of the annual US inpatient admissions, weighted samples (TRENDWT before 2011; DISCWT after 2012), stratum (NIS_STRATUM), and clusters (HOSPID) were used to generate national estimates for all the analyses. SAS software provides an analysis of sample survey data using the SURVEY procedure. Descriptive statistics of the included patients were presented as number (n) and weighted percentage (%), or mean and standard error (SE). Categorical data were analyzed by the PROC SURVEYFREQ method, and continuous data were analyzed by the PROC SURVEYREG method.

The patients included in the study were matched by age and sex using the propensity score matching (PSM) method, to reach a ratio of cases:controls = 1:4. Logistic regression analyses were used to calculate odds ratios (ORs) and 95% confidence intervals (CIs) for dichotomized outcomes. Variables with significant differences between the 2 comparison groups were entered into multivariable regression models for adjustments. All analyses were 2-sided, and a value of p < 0.05 was considered to indicate a statistically significant difference. All statistical analyses were performed using the statistical software package SAS version 9.4 (SAS Institute Inc., Cary, NC, US).

## Results

### Study population

**Figure 1:**
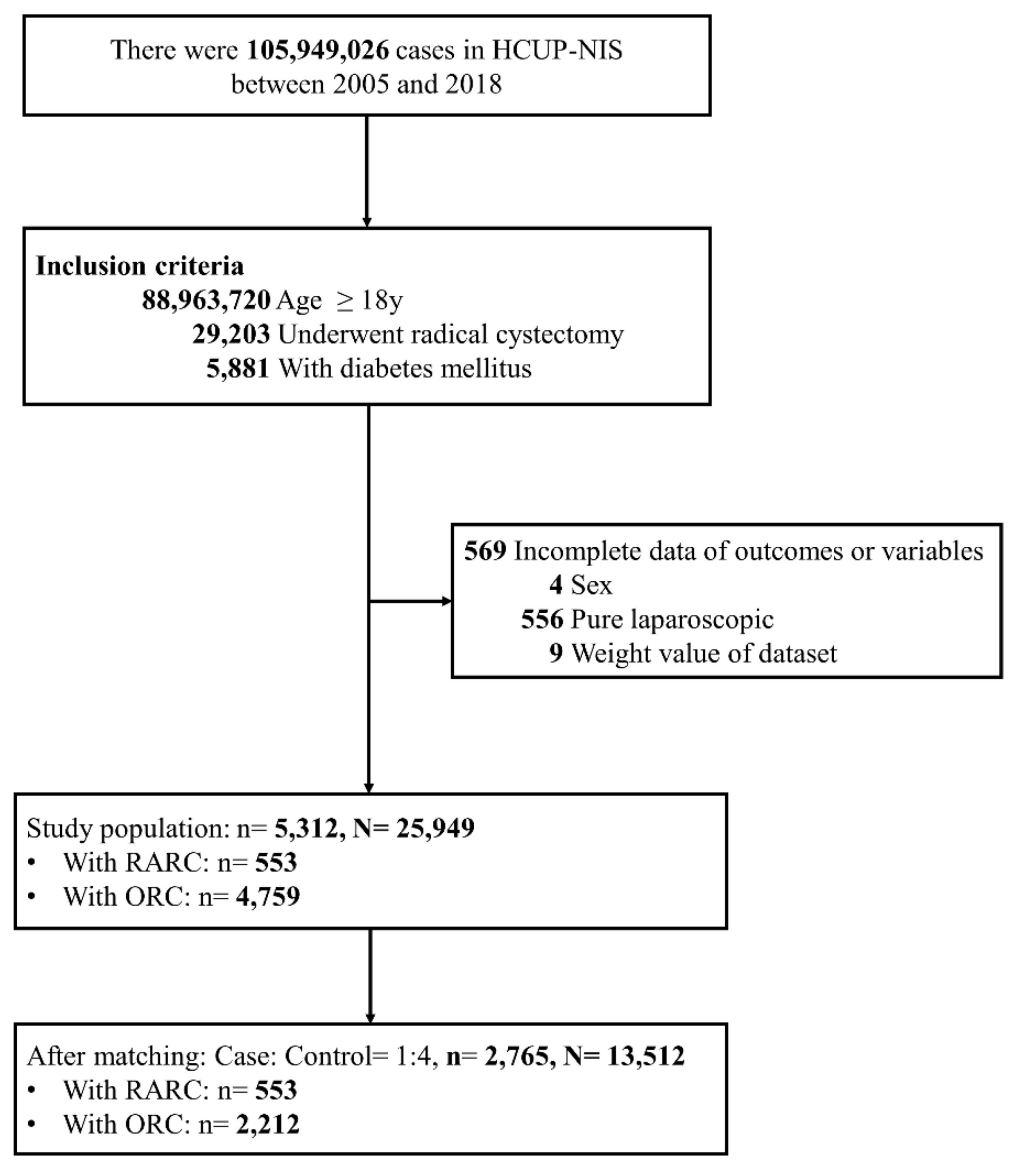
Flow diagram of patient selection and inclusion.

A flow diagram of patient selection and inclusion is shown in Figure 1. A total of 5,881 patients with DM who received a radical cystectomy between 2005 and 2018 were identified in the NIS database. Patients who received a pure laparoscopic radical cystectomy laparoscopic (n = 556) and those with missing information on study outcomes or variables (n = 13) were excluded. Finally, 5,312 patients were included as the study cohort. After PSM, 2,765 patients remained and were included in the analysis. This sample represented 13,512 US adults. Amongst, 553 (20.0%) patients underwent RARC and 2,212 (80.0%) underwent ORC.

### Characteristics of the study population after matching

Patient characteristics are summarized in Table 1. The mean age of the patients was 69 years, and 86% were males. Household income, lymph node invasion or metastatic disease, smoking, emergency admission, weekend admission, hospital bed size, and hospital location/ teaching status were significantly different between the 2 groups (all, p < 0.05).

### In-hospital outcomes after matching

In-hospital outcomes are summarized in Table 2. Patients who received RARC had lower percentages of urinary, wound, and device-related complications, prolonged LOS, and unfavorable discharge than patients who received ORC (all, p < 0.05).

### Associations between RARC and ORC and in-hospital outcomes

The relations between RACR and ORC and in-hospital outcomes are summarized in Table 3. After adjustment, patients who received RARC had a significantly lower risk of prolonged LOS (adjusted odd ratio (aOR) = 0.56, 95% CI: 0.45, 0.71), unfavorable discharge (aOR = 0.74, 95% CI: 0.56, 0.97), urinary complications (aOR = 0.75, 95% CI: 0.57, 0.98) and wound and device-related complications (aOR = 0.59, 95% CI: 0.41, 0.86) than patients who received ORC. Full analytic models are shown in Supplementary Tables S3-S4.

### Associations between RARC and ORC and in-hospital outcomes, stratified by age and Charlson Comorbidity Index (CCI)

The relations between RARC and ORC and in-hospital outcomes stratified by age and CCI are shown in Table 4. After adjustment, patients < 70 years old who received RARC were significantly less likely to have urinary complications (aOR = 0.59, 95% CI: 0.41, 0.84) and wound and device-related complications (aOR = 0.55, 95% CI: 0.32, 0.94) compared to those who received ORC. In patients with a CCI of 0-1, those that received RARC were significantly less likely to have urinary complications (aOR = 0.74, 95% CI: 0.56, 0.98) and wound and device-related complications (aOR = 0.63, 95% CI: 0.43, 0.93) compared to those that received ORC.

## Discussion

The present study used a nationally representative sample of the US to compare the outcomes of RARC and ORC in patients with bladder cancer and DM. The results showed that RARC is independently associated with a lower risk of prolonged LOS, unfavorable discharge, urinary complications, and wound and device-related complication than ORC.

The benefits of RARC are more prominent among patients < 70 years old and with a CCI score of 0-1 than in older patients. These results add further information to the literature which support the advantages of RARC over ORC, and specifically the advantages are seen in patients with DM.

DM has become a world-wide health concern, with a prevalence that is increasing yearly, in association with the obesity epidemic 2, 6, 9. The incidence of bladder cancer is also increasing 1, 2. Though some studies have found that type 2 DM is not associated with an increased risk of bladder cancer 9, many studies have shown that patients with DM have a higher risk of developing cancer in general, and specifically bladder cancer 10, 18. Notably, systematic reviews and meta-analyses have suggested that DM is associated with a poorer prognosis in patients with bladder cancer who undergo surgery (recurrence rate, cancer-specific survival) 19, 20. The negative impact of diabetes on bladder cancer outcomes, especially following surgeries like radical cystectomy, may be attributed to several mechanisms, including heightened inflammation and an increased susceptibility to infections 19, 20. As such, it is important to understand how the type of surgery and other factors influences the outcomes of patients with DM and bladder cancer who require surgery.

Radical cystectomy is the procedure of choice for patients with bladder cancer, and is a difficult procedure that can be associated with complications and marked morbidity. RARC simplifies the procedure and provides an improved surgical field compared to an open procedure 21. However, the learning curve is steep, and as a relatively new technology, it is important to determine how outcomes of RARC compare to that of the gold standard, ORC. A number of randomized controlled trials (RCTs) have compared RARC and ORC with respect to different outcomes, and the trials have indicated that RARC provides similar or better clinical and oncological outcomes as ORC 4, 14, 15.

In recent years, a number of literature reviews, and systematic reviews and meta-analyses have been published examining various outcomes of RARC compared to ORC. For example, a review of the literature by Iqbal *et al.* 22. published in 2021 suggested that functional outcomes are similar between RARC and ORC. In 2023, 2 systematic reviews and meta-analyses of randomized controlled trials compared RARC and ORC. Fontanet *et al.* 23. included 8 RCTs comprised of 1,024 patients and found that RARC is not inferior to ORC in terms of surgical safety and oncological outcomes, and RARC was associated with a lower blood transfusion rate. Liu *et al.* 24. also examined RCTs and reported that oncological outcomes, postoperative complications, and health-related quality of life were similar between the 2 procedures. An analysis of RCTs by Khetrapal *et al.* 25. found similar oncological outcomes between the 2 procedures, and that RARC was associated with less blood loss and a shorter hospital stay. An analysis by Kimura *et al.* 26. that included 6 RCTs and 31 non-randomized comparative studies no differences in quality of life score assessment, complications, length of hospital stay and mortality between the 2 procedures. In a unique study, Aminoltejari *et al.* 27. examined the data from current systematic reviews and meta-analyses. The analysis found that oncological outcomes and complications were similar between the 2 procedures, but quality of life outcomes required further study.

In contrast to previous literature that did not specifically address DM patients, our results indicate that the advantages of RARC appear to be even more pronounced in individuals with DM. Yuanming *et al.* 28. conducted a comprehensive analysis of risk factors for adverse perioperative outcomes in patients undergoing RARC. Notably, DM emerged as an independent risk factor associated with prolonged LOS. It is known that, DM patients, who contend with compromised immune responses and complex medical conditions, exhibit a heightened vulnerability to postoperative complications across various surgical settings 18. As mentioned previously, RARC, characterized by its minimally invasive approach, which entails smaller incisions, equips surgeons with enhanced precision and dexterity. This technique mitigates tissue trauma, reduces blood loss, and expedites the recovery process. For DM patients, these advantages contribute to improved wound healing, consequently positively impacting both wound complication rates and need for prolonged hospitalization. Therefore, it could provide larger benefit than in those without DM.

Finally, our investigations within the DM subgroup consistently demonstrated the superiority of RARC over ORC in several short-term outcomes. This held true even after rigorous control for various clinical factors, emphasizing the critical role of RARC in improving outcomes within this patient subgroup. Notably, our findings indicate that younger individuals (<70y), and those with lower comorbidity burdens, might gain even more substantial advantages from RARC in terms of short-term outcomes. This highlights that even in the population where surgery can be tolerated, RARC still emerges as a more favorable procedure.

### Strength and Limitations

The strength of the study is the analysis based on a very large, nationally representative sample. The results are likely generalizable to the entire population of the US. However, this study also has several limitations. First, due to the retrospective and observational design of the study, it's important to interpret the findings cautiously, recognizing the potential for selection bias. Second, errors in coding are possible, much like in prior claim-based studies that use the ICD code system. Third, the exact T stage was not recorded in the dataset, precluding our analysis. Also, the duration and treatment modality for DM was not available in the database. Additionally, the NIS database does not contain data on long-term follow-up, readmission rates, or survival after discharge, making it impossible to conduct an analysis. Lastly, although important, preoperative performance status and intraoperative characteristics such as type of urinary diversion, duration of the procedure, and amount of blood lost, were not taken into account in the analysis due to lack of data.

## Conclusions

In patients with DM and bladder cancer opting for surgery, compared to ORC, RARC appears to be associated with better short-term outcomes in terms of reduced risks for prolonged LOS, unfavorable discharge, urinary complication, as well as wound and device-related complications. Prospective studies are still warranted to further validate these findings.

## Supplementary Material

Supplementary tables.

## Figures and Tables

**Table 1:** Characteristics of the study population after matching

	Total (N = 2,765)	RARC (n = 553)	ORC (n = 2,212)	p-value
**Age, years**	69.4 ± 0.2	69.5 ± 0.3	69.4 ± 0.2	0.853
18-59	335 (12.1)	67 (12.2)	268 (12.1)	0.999
60-69	1040 (37.6)	208 (37.4)	832 (37.7)	
70-79	1060 (38.4)	212 (38.4)	848 (38.4)	
80+	330 (11.9)	66 (12.0)	264 (11.9)	
**Sex**				0.984
Male	2385 (86.3)	477 (86.3)	1908 (86.3)	
Female	380 (13.7)	76 (13.7)	304 (13.7)	
**Insurance status**				0.193
Medicare/Medicaid	1986 (71.9)	382 (69.3)	1604 (72.6)	
Private including HMO	704 (25.5)	152 (27.5)	552 (25.0)	
Self-pay/no-charge/other	71 (2.6)	18 (3.2)	53 (2.4)	
Missing	4	1	3	
**Household income**				**0.032**
Q1	639 (23.5)	113 (21.0)	526 (24.2)	
Q2	701 (25.8)	125 (23.0)	576 (26.5)	
Q3	739 (27.2)	163 (30.3)	576 (26.5)	
Q4	635 (23.5)	140 (25.8)	495 (22.9)	
Missing	51	12	39	
**Lymph node invasion or metastatic disease**				**0.036**
Yes	592 (21.3)	100 (18.0)	492 (22.2)	
No	2173 (78.7)	453 (82.0)	1720 (77.8)	
**Smoking**				**<0.001**
Yes	1167 (42.4)	272 (49.3)	895 (40.6)	
No	1598 (57.6)	281 (50.7)	1317 (59.4)	
**DM with end organ damage**				0.485
Yes	352 (12.8)	66 (11.9)	286 (13.0)	
No	2413 (87.2)	487 (88.1)	1926 (87.0)	
**CCI**				0.785
0-1	1965 (71.0)	389 (70.4)	1576 (71.1)	
2-3	686 (24.8)	144 (26.0)	542 (24.5)	
4-5	104 (3.8)	18 (3.3)	86 (4.0)	
6+	10 (0.4)	2 (0.4)	8 (0.4)	
**Emergent admission**				**<0.001**
Yes	277 (10.0)	23 (4.2)	254 (11.5)	
No	2482 (90.0)	530 (95.8)	1952 (88.5)	
Missing	6	0	6	
**Weekend admission**				**0.025**
Yes	140 (5.0)	17 (3.1)	123 (5.5)	
No	2625 (95.0)	536 (96.9)	2089 (94.5)	
**Hospital bed size**				**<0.001**
Small	301 (10.6)	98 (17.6)	203 (8.9)	
Medium	498 (18.3)	78 (14.3)	420 (19.3)	
Large	1956 (71.1)	376 (68.0)	1580 (71.8)	
Missing	10	1	9	
**Hospital location/ teaching status**				**<0.001**
Rural	50 (1.7)	3 (0.5)	47 (2.1)	
Urban nonteaching	444 (16.1)	54 (9.9)	390 (17.6)	
Urban teaching	2261 (82.2)	495 (89.6)	1766 (80.3)	
Missing	10	1	9	

Abbreviation: HMO, Health Maintenance Organization; RARC, robotic-assisted radical cystectomy; ORC, open radical cystectomy; DM, diabetes mellitus; and CCI, Charlson Comorbidity Index; Q, quartile. Continuous variables are presented as mean ± SE; categorical variables are presented as unweighted counts (weighted percentage). p-value < 0.05 shown in bold.

**Table 2:** In-hospital outcomes after matching

	Total (N = 2,765)	RARC (n = 553)	ORC (n = 2,212)	p-value
**In-hospital mortality**	46 (1.6)	11 (2.0)	35 (1.6)	0.459
**Complication, any**	1673 (60.5)	312 (56.2)	1361 (61.6)	**0.017**
AMI and cardiac complications	201 (7.3)	38 (6.8)	163 (7.4)	0.647
CVA and nervous system complications	65 (2.4)	12 (2.2)	53 (2.4)	0.721
VTE	94 (3.4)	13 (2.3)	81 (3.7)	0.105
Respiratory complications and pneumonia	370 (13.4)	68 (12.3)	302 (13.7)	0.381
Digestive system complications	531 (19.3)	95 (17.1)	436 (19.9)	0.155
Urinary complications	409 (14.8)	62 (11.3)	347 (15.7)	**0.005**
Vascular complication	28 (1.0)	4 (0.7)	24 (1.1)	0.360
Bleeding/transfusion	587 (21.2)	109 (19.6)	478 (21.5)	0.336
Infection	364 (13.2)	74 (13.3)	290 (13.2)	0.901
Sepsis/shock	292 (10.6)	57 (10.3)	235 (10.7)	0.787
Tracheostomy/mechanical ventilation	121 (4.3)	27 (4.8)	94 (4.2)	0.488
AKI	541 (19.7)	108 (19.6)	433 (19.7)	0.965
Wound and device-related complication	227 (8.2)	30 (5.4)	197 (8.9)	**0.003**
**Prolonged LOS ^a, b^**	780 (28.1)	103 (18.5)	677 (30.6)	**<0.001**
**Unfavorable discharge ^a^**	490 (18.0)	76 (14.1)	414 (19.0)	**0.008**

Abbreviation: RARC, robotic-assisted radical cystectomy; ORC, open radical cystectomy; AMI, acute myocardial infarction; CVA, cerebrovascular accident; VTE, venous thromboembolism; AKI, acute kidney injury; LOS, length of stay in hospital. Continuous variables are presented as mean ± SE; categorical variables are presented as unweighted counts (weighted percentage). ^a^ Excluding patients who died in the hospital. ^b^ LOS > 11 days. p-value < 0.05 shown in bold.

**Table 3:** Associations between RARC vs. ORC and in-hospital outcomes

Outcomes	Surgery	Univariable		Multivariable	
		OR (95% CI)	P-value	aOR (95% CI)	P-value
In-hospital mortality^ c^	RARC vs ORC	1.25 (0.69, 2.28)	0.460	1.48 (0.81, 2.69)	0.203
Prolonged LOS ^a, b, d^	RARC vs ORC	0.51 (0.41, 0.63)	**<0.001**	0.56 (0.45, 0.71)	**<0.001**
Unfavorable discharge ^a, d^	RARC vs ORC	0.70 (0.54, 0.91)	**0.008**	0.74 (0.56, 0.97)	**0.028**
Complication, any ^d^	RARC vs ORC	0.80 (0.67, 0.96)	**0.017**	0.83 (0.69, 1.01)	0.057
Infection^ d^	RARC vs ORC	1.02 (0.78, 1.33)	0.900	1.08 (0.83, 1.42)	0.568
Urinary complications^ d^	RARC vs ORC	0.68 (0.52, 0.89)	**0.005**	0.75 (0.57, 0.98)	0.037
AKI^ d^	RARC vs ORC	1.00 (0.80, 1.25)	0.965	1.04 (0.82, 1.31)	0.764
Wound and device-related complication^ d^	RARC vs ORC	0.58 (0.40, 0.84)	**0.004**	0.59 (0.41, 0.86)	**0.006**

Abbreviation: LOS, length of stay in hospital; AKI, acute kidney injury; OR, odd ratio; aOR, adjusted odd ratio; CI, confidence interval; CCI, Charlson Comorbidity Index. P-value < 0.05 is shown in bold. ^a^ Excluding patients who died in the hospital. ^b^ LOS > 8 days. ^c^ Adjusted for age group, smoking, CCI, and emergency admission. ^d^ Adjusted for age group, sex, insurance status, smoking, diabetes mellitus with end-organ damage, CCI, emergency admission, and weekend admission.

**Table 4:** Associations between RARC vs. ORC and in-hospital outcomes, stratified by age and CCI

Subgroup	Surgery	Infection		Urinary complications		AKI		Wound and device-related complication	
		aOR (95% CI)	P-value	aOR (95% CI)	P-value	aOR (95% CI)	P-value	aOR (95% CI)	P-value
**Age group**									
< 70	RARC vs. ORC	1.11 (0.77, 1.60)	0.575	0.59 (0.41, 0.84)	**0.003**	1.00 (0.73, 1.37)	0.989	0.55 (0.32, 0.94)	**0.029**
70+	RARC vs. ORC	1.04 (0.72, 1.50)	0.821	0.94 (0.66, 1.36)	0.751	1.08 (0.79, 1.47)	0.641	0.63 (0.39, 1.03)	0.064
**CCI**									
0-1	RARC vs. ORC	1.10 (0.84, 1.45)	0.492	0.74 (0.56, 0.98)	**0.034**	1.06 (0.84, 1.34)	0.633	0.63 (0.43, 0.93)	**0.018**
2+	RARC vs. ORC	0.54 (0.18, 1.60)	0.243	0.66 (0.21, 2.12)	0.470	0.73 (0.32, 1.67)	0.432	NA	

Abbreviation: AKI, acute kidney injury; RARC, robotic-assisted radical cystectomy; ORC, open radical cystectomy; CCI, Charlson Comorbidity Index; aOR, adjusted odd ratio; CI, confidence interval; NA, not applicable. P-value < 0.05 is shown in bold.
