# Supplementary material for: Short-term outcomes after robot-assisted versus open radical cystectomy for bladder cancer in patients with diabetes mellitus: an analysis of the United States Nationwide Inpatient Sample of 2005-2018
Source: Int J Med Sci. 2025 Jan 1;22(2):409–16. doi: 10.7150/ijms.102667 (PMC11704701; doi:10.7150/ijms.102667)
Supplement: Supplementary file 1 — Supplementary tables. [file ijmsv22p0409s1.pdf]

Supplemental Table S1. ICD codes used in the study

Diagnosis	ICD-9 code	ICD-10 code
**Undergoing radical cystectomy**	**PCS**: 57.7	**PCS**: 0TTB*
**Diabetes mellitus**	**CM**: 250	**CM**: E10-E14
**Pure laparoscopic**	**PCS**: 54.21, 54.51 but no 17.4	**PCS**: 0WJJ4ZZ, 0DNW4ZZ, 0TTB4ZZ, 0TTB7ZZ, 0TTB8ZZ but no 8E0W0CZ, 8E0W3CZ, 8E0W4CZ, 8E0W7CZ, 8E0W8CZ
**Robotic-assisted radical cystectomy**	**PCS**: 17.4	**PCS**: 8E0W0CZ, 8E0W3CZ, 8E0W4CZ, 8E0W7CZ, 8E0W8CZ
**AMI and cardiac complications**	**CM**: 410, 997.1, 785.51, 998.01, 427.5, 428.23, 458.29, 423.2, 423.3, 427.81, 426.0<br>**PCS**: 37.80<br>**DXCCS**: 100	**CM**: I21, I22, I97.7, I97.88, I97.89, R57.0, I46.9, I50.23, T81.11, I95.1, I31.3, I31.1, I31.4, I49.5, I44<br>**PCS**: 0JH60PZ, 0JH63PZ, 0JH80PZ, 0JH83PZ<br>DXCCSR_CIR009>0
**CVA and nervous system complications**	**CM**: 348.1, 430-437, 997.0<br>DXCCS: 109	**CM**: I60-I66, I67.0-I67.2, I67.4-I67.82, I67.841-I67.9, I68, I69, G45, G46, G93.1, G97<br>DXCCSR_CIR020>0
**VTE**	**CM**: 415, 451-453, 671, 673, 997.2	**CM**: I260, I269, I801-803, I808, I809, I820-I823, I828, I829, O082, O223, O871, O882, I81, I82
**Respiratory complications and pneumonia**	**CM**: 486, 481, 482.8, 482.3,997.3, 518.5, 518.8, 493.22, 512.1, 518.5, 518.81, 486, 481, 480, 482, 483, 484.7, 484.8, 4I5, 485, 514, 517, 073.0, 115.15, 115.95<br>DXCCS: 122	**CM**: A48.1, J12-J18, J95, J96, J80, J81.0, R06.03
**Digestive system complications**	**CM**: 567.22, 997.4, 998.59	**CM**: K91, K65.1, K66.0, K68.11
**Urinary complications**	**CM**: 595.0, 596.54, 598.9, 590.10, 599.0, 996.64, 788.20, 788.3, 997.5	**CM**: N99.1-N99.9, N39.0, R33.8, N31.1, N39.4, N35.1, N10, N30.00, N30.01, T83.510A, T83.511A, T83.512A, T83.518A
**Vascular complication**	**CM**: 997.2, 997.7, 444.2	**CM**: T81.7, I74.1, I74.2

Supplemental Table S1. ICD codes used in the study

Diagnosis	ICD-9 code	ICD-10 code
**Bleeding/ need for transfusion**	**CM**: V58.2, 998.1, 998.2, 998.51, 285.1, 459.0<br>**PCS**: 39.98, 99.0	**CM**: E87.71, J95.84, E83.111, T80.3, T80.4, T80.A, T80.5, T80.6<br>**PCS**: 0W380ZZ, 0W383ZZ, 0W384ZZ, 30233H, 30233J, 30233K, 30233L, 30233M, 30233N, 30233P, 30233R, 30233T, 30233V, 30233W
**Infection**	**CM**: 001-139, 998.5	**CM**: L00-L08, B99, T81.43, O86.03, T81.4
**Sepsis/shock**	**CM**: 995.9, 996.64, 038, 999.3, 790.7, 041, 785.52, 785.50, 785.52-785.59, 995.0, 038	**CM**: R78.81, A41, R65.2, T81.4, T80.2, A42.7, A22.7, B37.7, A26.7, A28.2, A54.86, B00.7, A32.7, A24.1, A39.2, A20.7, A21.7, A48.3, R57.1, R57.9, R55, T78.2, I97.1, T81.10, T81.12, T81.19, R65.1, A40, T82.6, T82.7, T85.71, T85.72, T85.73, T85.79, T83.5, T83.6, T84.5, T84.6, T84.7
**Tracheostomy/ mechanical ventilation**	**CM**: 216, 519.0<br>**PCS**: 96.7	**CM**: Z99.1, Z93.0<br>**PCS**: 5A19
**AKI**	**CM**: 584, V45.1<br>DXCCS: 157	**CM**: N17, N99.0<br>DXCCSR_GEN002>0
**Wound and device-related complication**	**CM**: 998.12, 998.13, 998.3, 998.83, 996.1, 996.62, 996.74, 998.2, 998.4, 998.7, 998.5, 998.2	**CM**: T81.3, T81.5, T81.6, K91.7
**Lymph node invasion or metastatic disease**	**CM**: 196-199	**CM**: C77-C79
**Smoking**	**CM**: 305.1, V15.82, 989.84	**CM**: Z71.6, Z72.0, Z86.43, Z87.891, F17, O99.33, T65.2
**DM with end organ damage**	**CM**: 250.4-250.7	**CM**: E10.2-E10.5, E10.7, E11.2-E11.5, E11.7, E12.2-E12.5, E12.7, E13.2-E13.5, E13.7, E14.2-E14.5, E14.7

Abbreviation: AMI, acute myocardial infarction; CVA, cerebrovascular accident; VTE, venous thromboembolism; AKI, acute kidney injury; DM, diabetes mellitus.

Supplementary Table 2. Characteristics of the study population before matching

	Total (N = 5,312)	RARC (n = 553)	ORC (n = 4,759)	P-value
**In-hospital mortality**	89 (1.7)	11 (2.0)	78 (1.6)	0.554
**Complication, any**	3256 (61.3)	312 (56.2)	2944 (61.9)	**0.011**
AMI and cardiac complications	449 (8.5)	38 (6.8)	411 (8.7)	0.137
CVA and nervous system complications	130 (2.5)	12 (2.2)	118 (2.5)	0.641
VTE	190 (3.6)	13 (2.3)	177 (3.8)	0.078
Respiratory complications and pneumonia	742 (14.0)	68 (12.3)	674 (14.2)	0.218
Digestive system complications	1001 (18.9)	95 (17.1)	906 (19.1)	0.289
Urinary complications	804 (15.2)	62 (11.3)	742 (15.6)	**0.005**
Vascular complication	54 (1.0)	4 (0.7)	50 (1.1)	0.394
Bleeding/transfusion	1132 (21.2)	109 (19.6)	1023 (21.4)	0.367
Infection	712 (13.4)	74 (13.3)	638 (13.4)	0.961
Sepsis/shock	573 (10.8)	57 (10.3)	516 (10.9)	0.665
Tracheostomy/mechanical Ventilation	232 (4.3)	27 (4.8)	205 (4.3)	0.533
AKI	1053 (20.0)	108 (19.6)	945 (20.0)	0.824
Wound and device-related complication	450 (8.5)	30 (5.4)	420 (8.8)	**0.003**
**Prolonged LOS [a, b]**	1539 (29.0)	103 (18.5)	1436 (30.2)	**<0.001**
**Unfavorable discharge [a]**	1030 (19.8)	76 (14.1)	954 (20.4)	**<0.001**
**Age, years)**	69.6 ± 0.1	69.5 ± 0.3	69.6 ± 0.1	0.758
18-59	734 (13.8)	67 (12.2)	667 (14.0)	0.075
60-69	1731 (32.6)	208 (37.4)	1523 (32.1)	
70-79	2162 (40.7)	212 (38.4)	1950 (41.0)	
80+	685 (12.9)	66 (12.0)	619 (13.0)	
**Sex**				**0.005**
Male	4358 (82.1)	477 (86.3)	3881 (81.6)	
Female	954 (17.9)	76 (13.7)	878 (18.4)	
**Insurance status**				0.051
Medicare/Medicaid	3900 (73.5)	382 (69.3)	3518 (74.0)	
Private including HMO	1252 (23.6)	152 (27.5)	1100 (23.1)	
Self-pay/no-charge/other	154 (2.9)	18 (3.2)	136 (2.9)	
Missing	6	1	5	
**Household income**				**0.003**
Q1	1264 (24.3)	113 (21.0)	1151 (24.7)	
Q2	1402 (26.9)	125 (23.0)	1277 (27.4)	
Q3	1346 (25.9)	163 (30.3)	1183 (25.4)	
Q4	1188 (22.9)	140 (25.8)	1048 (22.6)	
Missing	112	12	100	
**Lymph node invasion or metastatic disease**				**0.039**
Yes	1151 (21.6)	100 (18.0)	1051 (22.0)	
No	4161 (78.4)	453 (82.0)	3708 (78.0)	
**Smoking**				**<0.001**
Yes	2120 (40.1)	272 (49.3)	1848 (39.0)	

No	3192 (59.9)	281 (50.7)	2911 (61.0)	
**DM with end organ damage**				0.546
Yes	671 (12.7)	66 (11.9)	605 (12.8)	
No	4641 (87.3)	487 (88.1)	4154 (87.2)	
**CCI**				0.778
0-1	3818 (71.8)	389 (70.4)	3429 (71.9)	
2-3	1301 (24.6)	144 (26.0)	1157 (24.4)	
4-5	180 (3.4)	18 (3.3)	162 (3.4)	
6+	13 (0.2)	2 (0.4)	11 (0.2)	
**Emergent admission**				**<0.001**
Yes	553 (10.4)	23 (4.2)	530 (11.1)	
No	4750 (89.6)	530 (95.8)	4220 (88.9)	
Missing	9	0	9	
**Weekend admission**				0.099
Yes	242 (4.5)	17 (3.1)	225 (4.7)	
No	5070 (95.5)	536 (96.9)	4534 (95.3)	
**Hospital bed size**				**<0.001**
Small	558 (10.3)	98 (17.6)	460 (9.4)	
Medium	956 (18.3)	78 (14.3)	878 (18.8)	
Large	3771 (71.4)	376 (68.0)	3395 (71.8)	
Missing	27	1	26	
**Hospital location/ teaching status**				**<0.001**
Rural	131 (2.4)	3 (0.5)	128 (2.6)	
Urban nonteaching	874 (16.4)	54 (9.9)	820 (17.2)	
Urban teaching	4280 (81.2)	495 (89.6)	3785 (80.2)	
Missing	27	1	26	

Abbreviation: HMO, Health Maintenance Organization; RARC, robotic-assisted radical cystectomy; ORC, open radical cystectomy; AMI, acute myocardial infarction; CVA, cerebrovascular accident; VTE, venous thromboembolism; AKI, acute kidney injury; LOS, length of stay in hospital; DM, diabetes mellitus; CCI, Charlson Comorbidity Index.

Continuous variables are presented as mean ± SE; categorical variables are presented as unweighted counts (weighted percentage).

[a] Excluding patients died in the hospital.

[b] LOS > 11 days.

P-value < 0.05 is shown in bold.

Supplementary Table 3. Associations between study variables, in-hospital mortality, prolonged LOS, and unfavorable discharge

Variables	In-hospital mortality		Prolonged LOS [a]		Unfavorable discharge [a]	
	Univariable	Multivariable	Univariable	Multivariable	Univariable	Multivariable
	OR (95% CI)	aOR (95% CI)	OR (95% CI)	aOR (95% CI)	OR (95% CI)	aOR (95% CI)
**RARC vs. ORC**	1.25 (0.69, 2.28)	1.48 (0.81, 2.69)	**0.51 (0.41, 0.63)**	**0.56 (0.45, 0.71)**	**0.70 (0.54, 0.91)**	**0.74 (0.56, 0.97)**
**Age, years**						
18-59	Ref.	Ref.	Ref.	Ref.	Ref.	Ref.
60-69	0.93 (0.32, 2.71)	0.90 (0.31, 2.65)	1.08 (0.82, 1.42)	1.05 (0.79, 1.40)	0.82 (0.59, 1.15)	0.72 (0.50, 1.04)
70-79	2.07 (0.81, 5.30)	1.88 (0.75, 4.74)	**1.39 (1.06, 1.83)**	1.27 (0.94, 1.71)	**1.85 (1.33, 2.56)**	1.26 (0.86, 1.85)
80+	**4.69 (1.62, 13.56)**	**4.02 (1.41, 11.44)**	**1.51 (1.08, 2.10)**	1.31 (0.91, 1.88)	**4.31 (2.99, 6.23)**	**2.80 (1.84, 4.25)**
**Sex**						
Male	1.35 (0.55, 3.36)		**0.79 (0.64, 0.99)**	0.80 (0.63, 1.00)	**0.54 (0.42, 0.68)**	**0.58 (0.45, 0.75)**
Female	Ref.		Ref.	Ref.	Ref.	Ref.
**Insurance status**						
Medicare/Medicaid	Ref.		Ref.	Ref.	Ref.	Ref.
Private including HMO	0.94 (0.52, 1.71)		**0.77 (0.64, 0.92)**	0.96 (0.79, 1.18)	**0.26 (0.20, 0.35)**	**0.39 (0.28, 0.54)**
Self-pay/no-charge/ other	1.67 (0.61, 4.59)		0.76 (0.46, 1.26)	0.97 (0.55, 1.72)	0.60 (0.31, 1.16)	0.84 (0.43, 1.65)
**Household income**						
Q1	1.19 (0.58, 2.45)		1.22 (0.97, 1.53)		1.12 (0.86, 1.47)	
Q2	0.90 (0.40, 2.02)		1.03 (0.81, 1.31)		0.90 (0.69, 1.19)	
Q3	1.19 (0.59, 2.39)		1.04 (0.82, 1.31)		0.90 (0.69, 1.18)	
Q4	Ref.		Ref.		Ref.	
**Lymph node invasion or metastatic disease**						
Yes	0.44 (0.17, 1.12)		1.18 (0.97, 1.42)		1.08 (0.86, 1.36)	
No	Ref.		Ref.		Ref.	
**Smoking**						
Yes	**0.46 (0.25, 0.84)**	**0.49 (0.27, 0.91)**	**0.65 (0.55, 0.77)**	**0.68 (0.57, 0.81)**	**0.77 (0.64, 0.94)**	0.85 (0.69, 1.04)
No	Ref.		Ref.	Ref.	Ref.	Ref.
**DM with end-organ damage**						
Yes	1.66 (0.86, 3.20)		**1.31 (1.04, 1.66)**	1.01 (0.78, 1.32)	**1.81 (1.42, 2.30)**	**1.31 (1.00, 1.73)**
No	Ref.		Ref.	Ref.	Ref.	Ref.
**CCI**						
0-1	Ref.	Ref.	Ref.	Ref.	Ref.	Ref.
2-3	**1.97 (1.14, 3.42)**	**1.90 (1.09, 3.30)**	**1.60 (1.34, 1.91)**	**1.65 (1.36, 2.00)**	**1.82 (1.49, 2.23)**	**1.65 (1.32, 2.06)**
4-5	0.64 (0.09, 4.73)	0.57 (0.07, 4.33)	**2.87 (1.95, 4.23)**	**2.76 (1.82, 4.17)**	**2.66 (1.76, 4.02)**	**2.14 (1.36, 3.38)**
6+	**8.60 (1.07, 69.24)**	10.34 (0.90, 118.35)	0.35 (0.04, 2.76)	0.34 (0.03, 3.32)	1.55 (0.33, 7.23)	2.10 (0.38, 11.77)
**Emergent admission**						
Yes	**0.34 (0.18, 0.64)**	**0.36 (0.19, 0.68)**	**0.35 (0.28, 0.45)**	**0.40 (0.32, 0.52)**	**0.52 (0.40, 0.68)**	**0.58 (0.44, 0.76)**
No	Ref.	Ref.	Ref.	Ref.	Ref.	Ref.
**Weekend admission**						
Yes	Ref.		Ref.	Ref.	Ref.	Ref.
No	0.61 (0.22, 1.73)		**0.42 (0.30, 0.57)**	**0.55 (0.39, 0.78)**	**0.61 (0.42, 0.87)**	0.79 (0.52, 1.19)
**Hospital bed size**						
Small	1.60 (0.84, 3.05)		0.92 (0.75, 1.13)		0.87 (0.65, 1.15)	
Medium	1.54 (0.82, 2.88)		0.93 (0.76, 1.13)		1.13 (0.89, 1.43)	
Large	Ref.		Ref.		Ref.	
**Hospital location/teaching status**						
Rural	**1.40 (1.01, 1.95)**		0.78 (0.49, 1.26)		0.70 (0.35, 1.37)	
Urban nonteaching	1.47 (0.83, 2.61)		1.13 (0.93, 1.39)		1.12 (0.89, 1.41)	
Urban teaching	Ref.		Ref.		Ref.	

Abbreviation: HMO, Health Maintenance Organization; RARC, robotic-assisted radical cystectomy; ORC, open radical cystectomy; LOS, length of hospital stay; DM, diabetes mellitus; CCI, Charlson Comorbidity Index; OR, odd ratio; aOR, adjusted odds ratio; CI, confidence interval.

P-value < 0.05 is shown in bold.

[a] Excluding patients who died in the hospital.

Variables that were significant in univariable regression were adjusted in multivariable models.

Supplementary Table 4. Associations between study variables and postoperative complications

Variables	Complication, any		Infection		Urinary complications		AKI		Wound and device-related complication	
	Univariable	Multivariable	Univariable	Multivariable	Univariable	Multivariable	Univariable	Multivariable	Univariable	Multivariable
	OR (95% CI)	aOR (95% CI)	OR (95% CI)	aOR (95% CI)	OR (95% CI)	aOR (95% CI)	OR (95% CI)	aOR (95% CI)	OR (95% CI)	aOR (95% CI)
**RARC vs. ORC**	**0.80 (0.67, 0.96)**	0.83 (0.69, 1.01)	1.02 (0.78, 1.33)	1.08 (0.83, 1.42)	**0.68 (0.52, 0.89)**	**0.75 (0.57, 0.98)**	1.00 (0.80, 1.25)	1.04 (0.82, 1.31)	**0.58 (0.40, 0.84)**	**0.59 (0.41, 0.86)**
**Age, years**										
18-59	Ref.	Ref.	Ref.	Ref.	Ref.	Ref.	Ref.	Ref.	Ref.	Ref.
60-69	0.89 (0.70, 1.14)	0.85 (0.66, 1.10)	1.00 (0.71, 1.40)	0.95 (0.67, 1.35)	**0.56 (0.42, 0.76)**	**0.56 (0.41, 0.75)**	1.22 (0.90, 1.67)	1.12 (0.81, 1.54)	0.92 (0.60, 1.43)	0.91 (0.59, 1.40)
70-79	1.12 (0.88, 1.44)	0.97 (0.73, 1.29)	1.01 (0.72, 1.43)	0.85 (0.59, 1.23)	**0.57 (0.42, 0.77)**	**0.52 (0.37, 0.71)**	1.31 (0.97, 1.79)	1.04 (0.75, 1.45)	0.98 (0.64, 1.49)	0.86 (0.56, 1.31)
80+	1.32 (0.98, 1.79)	1.10 (0.79, 1.53)	0.89 (0.58, 1.38)	0.73 (0.46, 1.15)	0.83 (0.58, 1.19)	0.69 (0.47, 1.02)	**1.58 (1.09, 2.29)**	1.33 (0.88, 1.99)	1.19 (0.71, 1.99)	1.01 (0.60, 1.69)
**Sex**										
Male	**0.66 (0.54, 0.82)**	**0.65 (0.52, 0.81)**	0.80 (0.59, 1.08)	0.80 (0.59, 1.08)	**0.51 (0.39, 0.66)**	**0.54 (0.42, 0.71)**	1.16 (0.88, 1.52)	1.15 (0.87, 1.52)	**0.67 (0.47, 0.94)**	**0.68 (0.48, 0.96)**
Female	Ref.	Ref.	Ref.	Ref.	Ref.	Ref.	Ref.	Ref.	Ref.	Ref.
**Insurance status**										
Medicare/Medicaid	Ref.	Ref.	Ref.	Ref.	Ref.	Ref.	Ref.	Ref.	Ref.	Ref.
Private including HMO	**0.77 (0.65, 0.91)**	0.90 (0.74, 1.10)	0.80 (0.62, 1.03)	0.77 (0.58, 1.02)	0.92 (0.73, 1.17)	0.88 (0.67, 1.15)	**0.79 (0.64, 0.97)**	0.95 (0.75, 1.21)	0.73 (0.54, 1.00)	0.76 (0.54, 1.06)
Self-pay/no-charge/ other	1.13 (0.71, 1.78)	1.39 (0.86, 2.25)	1.04 (0.55, 1.96)	1.09 (0.58, 2.07)	0.82 (0.44, 1.52)	0.84 (0.45, 1.56)	1.42 (0.89, 2.26)	**1.92 (1.21, 3.04)**	1.29 (0.64, 2.61)	1.45 (0.70, 2.99)
**Household income**										
Q1	1.11 (0.90, 1.37)		0.75 (0.55, 1.03)		1.00 (0.76, 1.32)		1.14 (0.87, 1.50)		1.10 (0.71, 1.69)	
Q2	1.05 (0.86, 1.29)		0.95 (0.71, 1.27)		1.04 (0.79, 1.38)		1.30 (1.00, 1.69)		1.15 (0.78, 1.70)	
Q3	1.07 (0.87, 1.31)		1.02 (0.76, 1.36)		1.08 (0.82, 1.42)		1.09 (0.84, 1.42)		0.99 (0.67, 1.45)	
Q4	Ref.		Ref.		Ref.		Ref.		Ref.	
**Lymph node invasion or metastatic disease**										
Yes	1.02 (0.85, 1.21)		0.98 (0.75, 1.28)		1.00 (0.77, 1.30)		1.17 (0.94, 1.45)		**1.61 (1.21, 2.15)**	
No	Ref.		Ref.		Ref.		Ref.		Ref.	
**Smoking**										
Yes	**0.86 (0.74, 1.00)**	0.87 (0.74, 1.01)	**0.74 (0.60, 0.92)**	**0.73 (0.58, 0.91)**	**0.80 (0.65, 0.98)**	0.83 (0.67, 1.03)	0.93 (0.77, 1.11)	0.87 (0.72, 1.05)	**0.69 (0.53, 0.91)**	**0.71 (0.54, 0.94)**
No	Ref.	Ref.	Ref.	Ref.	Ref.	Ref.	Ref.	Ref.	Ref.	Ref.
**DM with end-organ damage**										
Yes	**1.72 (1.36, 2.17)**	1.20 (0.94, 1.55)	1.23 (0.90, 1.68)	1.06 (0.74, 1.50)	1.26 (0.96, 1.65)	1.08 (0.80, 1.45)	**2.27 (1.80, 2.87)**	1.29 (0.99, 1.69)	1.00 (0.67, 1.49)	0.84 (0.55, 1.28)
No	Ref.	Ref.	Ref.	Ref.	Ref.	Ref.	Ref.	Ref.	Ref.	Ref.
**CCI**										
0-1	Ref.	Ref.	Ref.	Ref.	Ref.	Ref.	Ref.	Ref.	Ref.	Ref.
2-3	**2.34 (1.93, 2.84)**	**2.32 (1.91, 2.83)**	**1.43 (1.14, 1.81)**	**1.43 (1.11, 1.84)**	**1.38 (1.11, 1.72)**	**1.44 (1.13, 1.82)**	**4.26 (3.50, 5.19)**	**4.11 (3.33, 5.08)**	1.27 (0.95, 1.70)	1.34 (0.98, 1.84)
4-5	**3.55 (2.26, 5.58)**	**3.25 (2.05, 5.14)**	1.57 (0.93, 2.65)	1.49 (0.86, 2.58)	1.16 (0.70, 1.93)	1.06 (0.62, 1.81)	**6.22 (4.22, 9.19)**	**5.90 (3.94, 8.84)**	1.60 (0.85, 3.00)	1.62 (0.86, 3.04)
6+	1.92 (0.59, 6.21)	1.98 (0.56, 6.99)	1.94 (0.42, 8.96)	2.03 (0.40, 10.33)	0.73 (0.09, 5.70)	0.78 (0.09, 6.84)	**7.04 (2.15, 23.01)**	**7.17 (2.21, 23.31)**	1.21 (0.15, 9.47)	1.44 (0.17, 11.90)
**Emergent admission**										
Yes	**0.57 (0.44, 0.74)**	**0.63 (0.48, 0.82)**	**0.77 (0.56, 1.07)**	0.82 (0.59, 1.14)	**0.43 (0.33, 0.57)**	**0.49 (0.37, 0.65)**	**0.64 (0.49, 0.83)**	**0.62 (0.47, 0.82)**	0.90 (0.58, 1.39)	1.04 (0.67, 1.61)

Supplementary Table 4. Associations between study variables and postoperative complications

Variables	Complication, any		Infection		Urinary complications		AKI		Wound and device-related complication	
	Univariable	Multivariable	Univariable	Multivariable	Univariable	Multivariable	Univariable	Multivariable	Univariable	Multivariable
	OR (95% CI)	aOR (95% CI)	OR (95% CI)	aOR (95% CI)	OR (95% CI)	aOR (95% CI)	OR (95% CI)	aOR (95% CI)	OR (95% CI)	aOR (95% CI)
No	Ref.	Ref.	Ref.	Ref.	Ref.	Ref.	Ref.	Ref.	Ref.	Ref.
**Weekend admission**										
Yes	Ref.	Ref.	Ref.	Ref.	Ref.	Ref.	Ref.	Ref.	Ref.	Ref.
No	**0.56 (0.40, 0.80)**	**0.64 (0.45, 0.92)**	0.74 (0.48, 1.14)	0.79 (0.51, 1.24)	**0.48 (0.33, 0.71)**	**0.58 (0.39, 0.86)**	0.99 (0.67, 1.46)	1.16 (0.76, 1.79)	0.73 (0.42, 1.26)	0.79 (0.45, 1.41)
**Hospital bed size**										
Small	1.02 (0.81, 1.28)		0.85 (0.64, 1.13)		1.09 (0.84, 1.41)		**1.26 (1.00, 1.58)**		0.77 (0.51, 1.14)	
Medium	1.03 (0.85, 1.24)		0.69 (0.52, 0.91)		1.03 (0.79, 1.34)		1.12 (0.89, 1.41)		0.87 (0.63, 1.20)	
Large	Ref.		Ref.		Ref.		Ref.		Ref.	
**Hospital location/teaching status**										
Rural	0.87 (0.58, 1.31)		0.68 (0.37, 1.24)		**0.50 (0.45, 0.56)**		0.94 (0.54, 1.63)		1.02 (0.57, 1.81)	
Urban nonteaching	1.14 (0.94, 1.38)		1.03 (0.79, 1.35)		1.11 (0.87, 1.42)		0.98 (0.78, 1.22)		1.14 (0.83, 1.56)	
Urban teaching	Ref.		Ref.		Ref.		Ref.		Ref.	

Abbreviation: HMO, Health Maintenance Organization; RARC, robotic-assisted radical cystectomy; ORC, open radical cystectomy; DM, diabetes mellitus; CCI, Charlson Comorbidity Index; OR, odd ratio; aOR, adjusted odds ratio; CI, confidence interval.
P-value < 0.05 is shown in bold.
Variables that were significant in univariable regression were adjusted in multivariable models.
